# Effects of Milling Methods on the Physicochemical Properties of Rice Flour from Indica, Japonica, and Glutinous Rice

**DOI:** 10.3390/foods15020275

**Published:** 2026-01-12

**Authors:** Chunlei Zheng, Zhenzhen Ren, Limin Li, Xueling Zheng

**Affiliations:** 1 College of Biological Engineering, Henan University of Technology, Zhengzhou 450001, China; zcl128@stu.haut.edu.cn; 2 College of Food Science and Engineering, Henan University of Technology, Zhengzhou 450001, China; rzz0038@163.com

**Keywords:** rice flour, milling methods, physicochemical properties

## Abstract

This study evaluated the effects of three milling methods, which are dry, semi-dry, and wet milling, on the physicochemical, thermal, and rheological properties of three types of broken rice (indica, japonica, and glutinous rice). The aim was to evaluate how these milling methods affect key flour characteristics, including starch damage, particle size distribution, swelling power, solubility, and gelatinization behavior. Dry milling resulted in the highest degree of starch damage, leading to increased solubility and swelling power, but also a reduction in gelatinization temperature and paste viscosity. Semi-dry milling exhibited moderate starch damage, enhanced thermal stability, and superior functional properties in comparison to dry milling. Wet milling, while minimizing starch damage, produced finer particles but resulted in lower swelling power and solubility. The results also indicated that Japonica rice exhibited the least starch damage, followed by Indica and Glutinous rice. These findings provide important insights into optimizing milling techniques for high-quality rice flour production, particularly for gluten-free food products. Overall, milling method substantially modulates structure and function relations in rice flour, and semi-dry and wet milling preserve starch integrity better than dry milling. These results provide practical guidance for selecting milling strategies to tailor flour functionality for specific rice-based products.

## 1. Introduction

Rice flour has long been a staple ingredient in many traditional and modern meals, including gluten-free products such as noodles, cakes, and rice-based snacks. The texture, consistency, and sensory attributes of flour largely depend on the milling process, which plays a vital role in shaping the physical and chemical characteristics of the flour [1,2]. Different rice varieties exhibit distinct starch compositions: indica rice typically has higher amylose content, resulting in firmer textures, while japonica rice contains moderate amylose, contributing to a stickier texture [3,4]. Glutinous rice, which is almost free of amylose, is composed almost entirely of amylopectin, leading to a highly cohesive, sticky starch gel [5,6]. These starch differences influence how rice flour behaves during processing, such as in noodle production, where milling methods can further modify these properties. Milling method selection significantly impacts key rice flour properties, including particle size distribution, starch damage, gelatinization, and pasting behavior [7,8]. Dry milling, the most commonly used industrial method, involves grinding rice without water; it is simple and has the advantage of low water consumption. However, it induces substantial starch damage, which increases solubility and swelling power but reduces gelatinization and adversely affects final product texture [7,9]. Wet milling, where rice is soaked before grinding, reduces starch damage by preserving granule integrity. However, its high water usage and wastewater generation raise sustainability concerns [9,10]. Semi-dry milling, a hybrid of dry and wet methods, has emerged as a viable alternative. By adding controlled moisture to rice before grinding, semi-dry milling reduces starch damage while minimizing water consumption compared to wet milling. Semi-dry milling preserves many beneficial properties of wet milling, such as reduced starch damage and improved gelatinization, while offering lower water usage and environmental impact [8]. Additionally, it provides better control over particle size distribution, which is critical for optimizing rheological properties and achieving desired textures in rice noodles [11,12].

The physicochemical properties of rice flour, including starch crystallinity, swelling power, solubility, and pasting behavior, are crucial for determining its suitability in food applications. These properties are influenced by both rice variety and milling method. For example, rice flour from Japonica varieties generally exhibits higher amylose content compared to Indica varieties, influencing its gelatinization and pasting characteristics [12]. Moreover, the degree of starch damage induced by milling processes significantly alters the functional properties of the flour. For instance, higher levels of damaged starch tend to lower the gelatinization temperature, decrease paste viscosity, and enhance the flour’s water-retention capacity, which, in turn, affects the texture and stability of products such as noodles [2,13,14,15].

Nonetheless, most prior work has focused on one rice variety or compared only two methods. Few studies systematically examine all three milling approaches across different subspecies or on broken rice. Broken rice, a byproduct from milling, is commonly used for flour production, but its behavior under different milling conditions is under-researched. In particular, there is a knowledge gap regarding how dry, semi-dry, and wet milling differentially affect the compositional, thermal, and pasting properties of flours from indica, japonica, and glutinous rice.

Given the growing demand for rice flour in gluten-free products and the increasing emphasis on sustainable processing methods, understanding the impact of different milling techniques on rice flour properties is crucial. This study aims to compare the effects of dry, semi-dry, and wet milling on the physicochemical, thermal, and rheological properties of rice flours from Japonica, Indica, and glutinous rice varieties. The findings from this research are expected to provide valuable insights into optimizing milling strategies for producing high-quality rice flour suitable for gluten-free food applications.

## 2. Materials and Methods

Indica, japonica, and glutinous broken rice were collected from a rice factory in Zhengzhou, China.

### 2.1. Preparation of Rice Flour

Three rice flour samples (japonica, Indica, and glutinous rice) were prepared: dry-milled flour (DMF), semi-dry-milled flour (S-DMF), and wet-milled flour (WMF).

For each milling experiment, a starting mass of 100 g of broken rice was used. The fundamental parameters of the three rice varieties are shown in Table 1.

Preparation of DMF: Milled rice was ground using an Ultrafine Pulverizer (ZN-08, Beijing Xingshilihe Technology Development Co., Ltd., Beijing, China). Flour samples (japonica, Indica, and glutinous rice) were passed through an 80-mesh sieve (0.2 mm opening), sealed in polypropylene plastic bags, and stored in a desiccator at room temperature until further analyses.

Preparation of S-DMF: The rice flour samples (japonica, Indica, and glutinous rice) were fully soaked in distilled water at a ratio of 1:3. After immersion, the remaining water was poured out, and the volume of the poured water was recorded. The difference between the volume of soaking water and the volume of residual water is the maximum water absorption. After the rice flour samples were quenched to the maximum water absorption rate, they were ground using an Ultrafine Pulverizer (ZN-08, Beijing Xingshilihe Technology Development Co., Ltd., Beijing, China) and sifted through an 80-mesh sieve (0.2 mm opening). The rice flour was dried in a hot air oven (101-0AB, Tianjin Taisite Instrument Co., Ltd., Tianjin, China) at 40 °C to obtain a moisture content of approximately 10%.

Preparation of WMF: Mix rice flour samples (japonica rice, Indica rice, and glutinous rice) with water in a 1:2 ratio. Place the soaked rice fragments into a colloid mill, adding water equivalent to 55% of the rice mass. Grind in the colloid mill until the rice paste exhibits no discernible granular texture. Proceed with vacuum filtration followed by freeze-drying. Subsequently, the milled rice flour undergoes secondary grinding using a colloid mill (Model JMS-50D, Langfang Langtong Machinery Co., Ltd., Langfang, China). The flour samples are sieved through an 80-mesh sieve with a 0.2 mm aperture and stored in a desiccator.

### 2.2. Determination of Basic Components of Rice Flour

Moisture content was analyzed with reference to AACC44-19 [16]. Starch content was analyzed by measuring the optical rotation values of HCl-treated hydrolysates, according to national standard GB/T 20378-2006 [17]. Damaged starch content was measured via the enzymatic colorimetric method with a starch damage assay kit (K-SDAM, Megazyme International Ltd., Wicklow, Ireland). Crude protein content was analyzed via the Kjeldahl method. The conversion factor of rice protein was 5.95. Ash content was analyzed on the basis of AACC 08-01 [18].

### 2.3. Particle Size Distribution of Rice Flour 

The particle size distribution was analyzed by a Laser Light Scattering Particle Size Analyzer (BT-9300H, Bettersize Instruments Ltd., Dandong, China) according to a previously published method [19].

### 2.4. Scanning Electron Microscopy of Rice Flour

For scanning electron microscopy (SEM) analysis, a dried sample was uniformly dispersed on a double-sided conductive adhesive, and then the rice flour samples were sputter-coated with gold-palladium to render them electrically conductive. Samples were then examined, and images were recorded with a scanning electron microscope (Quanta 250 FEG, FEI, Ted Pella, Redding, CA, USA) at an accelerating voltage of 13 kV. Samples were observed at magnification levels of 5000×.

### 2.5. Solubility and Swelling of Rice Flour

One gram of rice flour (dry basis, m_0_ = 1.00 g) was weighed into a 50-mL centrifuge tube and brought to a 5% (*w*/*w*) suspension by adding distilled water. The suspensions were incubated in a constant-temperature orbital shaker at 50, 60, 70, 80, and 90 °C for 30 min, then centrifuged at 3000 rpm for 20 min. The supernatant was carefully decanted and dried to constant weight in an oven at 105 °C; the dried weight of soluble solids was recorded as A (g). The wet sediment remaining in the centrifuge tube was weighed and recorded as P (g) [9]. Solubility and swelling power at each temperature were calculated as follows:

(1) $$Solubility\;\left(\%\right)=\frac{A}{m_{0}}\times 100$$

(2) $$Swelling\;power=\frac{P}{\left(m_{0}-A\right)}$$

where m_0_ is the initial dry weight of sample (g), A is the mass of dried soluble solids obtained from the supernatant (g), and P is the wet mass of the sediment after centrifugation (g).

### 2.6. Determination of Pasting Properties

The pasting properties of the rice flours were determined using a Brabender Viscoamylograph (Brabender GmbH & Co. KG, Duisburg, Germany) according to a method described by Ye et al. [20] with slight modifications. Briefly, a rice flour suspension was prepared by dispersing a sample quantity equivalent to 40 g on a 14% moisture basis in 360 g of distilled water. The suspension was subjected to a controlled heating and cooling cycle: it was heated from 35 °C to 95 °C at a rate of 1.5 °C/min, held at 95 °C for 30 min, then cooled to 50 °C at the same rate, and finally held at 50 °C for 30 min. Parameters, including pasting temperature, peak viscosity, breakdown, and setback, were recorded from the pasting curve.

### 2.7. X-Ray Diffraction (XRD) Measurement of Rice Flour

XRD was performed via an X-ray diffractometer (D8 Discover, Bruker, AXS, Karlsruhe, Germany). Prior to measurement, samples were equilibrated for 24 h in a desiccator over a saturated NaCl solution to ensure moisture equilibrium. Diffraction data were collected using Cu Kα radiation (λ = 1.54184 Å) with an operating current of 40 mA and an accelerating voltage of 40 kV (generator power 1400 W). Scans were performed over a 2θ range of 4–40° at a scan rate of 4° min^−1^ with a step size of 0.02 [21]. The relative crystallinity was calculated from the diffractograms by determining the ratio of the area under the crystalline peaks to the total area (crystalline + amorphous).

### 2.8. Determination of the Thermal Properties of Rice Flour

Thermal stability of rice flours produced by different milling methods was evaluated using a differential scanning calorimeter (PerkinElmer DSC 8500, USA). The method is based on the reference document with minor modifications [22]. Approximately 2.5 mg of oven-dry rice flour (dry basis) was accurately weighed into an aluminum DSC pan, and 7.5 µL (0.0075 mL) of distilled water was added. The pan was hermetically sealed and equilibrated at ambient temperature for 24 h prior to analysis. DSC scans were performed from 20 °C to 120 °C at a heating rate of 10 °C·min^−1^. An empty hermetically sealed aluminum pan was used as the reference.

### 2.9. Determination of the Rheological Properties of Rice Flour

Dynamic rheological properties of rice flour suspensions prepared from different milling methods were measured using a Haake RS6000 rheometer (Thermo Fisher Scientific, Drei Eichen, Hesse, Germany). Following a modified procedure based on reference [23], rice flour suspensions (10% *w*/*w*) were prepared and stirred on a magnetic stirrer for 1 h to ensure homogeneity. Aliquots (1.0 mL) of the suspension were transferred to the rheometer and tested using a parallel-plate geometry (PP35Ti rotor) with a gap of 1.0 mm. Temperature sweeps were conducted in strain-controlled mode: the samples were heated from 25 °C to 90 °C at 3.25 °C·min^−1^, held at 90 °C for 10 min, and then cooled to 25 °C at the same rate. The applied strain amplitude was 1% and the frequency was 1 Hz.

### 2.10. Statistical Analysis

The data were analyzed using SPSS (version 13.0 for Windows, SPSS Inc., Chicago, IL, USA). Statistical significance was assessed by analysis of variance followed by Duncan’s multiple range test at *p* < 0.05.

## 3. Results and Discussion

### 3.1. Properties of Rice Flour

Table 2 presents the effects of different milling methods on the basic components of indica, japonica, and glutinous rice flours. Both indica and glutinous rice flours had lower ash contents than japonica flour, while glutinous rice flour displayed the highest whiteness. These differences are likely due to the intrinsic characteristics of the rice varieties, with glutinous rice’s high amylopectin content contributing to its greater whiteness. Among the milling methods, WMF consistently produced rice flour with the lowest moisture and ash content, as well as the highest whiteness. The wet milling process effectively removes surface dust and bran, which lowers ash content and increases whiteness [24]. Protein content showed no significant differences across methods, while crude starch content followed the trend: DMF > S-DMF > WMF. This is primarily due to water loss during semi-dry and wet milling, which reduces the starch content on a dry-weight basis. Udomrati et al. [25] similarly reported that wet-milled rice flour had a significantly higher whiteness index than dry-milled flour, whereas the dry-milled powder was light brown due to heat-induced browning. In short, wet milling produces purer flour with better visual quality.

### 3.2. Particle Size Distribution of Rice Flour

The presence of fine and uniform rice vermicelli particles is the primary indicator of the edibility of rice vermicelli. The particle size distribution of rice vermicelli produced by different milling processes is shown in Table 3. Particle size distribution of rice flour varied significantly across both rice varieties and milling methods. Glutinous rice flour had the smallest particles, followed by japonica and indica. This aligns with the understanding that higher protein content, as found in indica rice, results in harder kernels and coarser flour. This is a consequence of the continuity of the protein matrix in which the starch granules are embedded, and because of this continuity and the absence of air spaces between the starch granules, the hardness of the endosperm is greater, which contributes to greater resistance to deformation forces during milling, and consequently to larger particles of the ground material. WMF produced the finest particles, while S-DMF resulted in the largest particles. The intense mechanical force of DMF also produced smaller particles. In contrast, S-DMF, which involves tempering with water, increased grain toughness and resulted in coarser particles. Thus, the ranking of average particle size was roughly: WMF < DMF < S-DMF for each variety. Other studies report a similar trend: for example, Udomrati, Tungtrakul, Lowithun, and Thirathumthavorn [25] observed that dry-milled flour had the largest particle size and wet-milled flour the smallest. Finer particles generally enhance gelatinization and textural properties; indeed, small, uniform starch granules are known to produce stronger gel networks and improved product texture.

### 3.3. Gelatinization Properties of Rice Flour

The predominant composition of rice is starch, with viscosity characteristics serving as a pivotal metric for evaluating its quality. It is imperative to ascertain the viscosity properties of rice flour in order to evaluate its quality. The apparent viscosity of a solution is chiefly reflected by the capacity of starch granules to absorb water and subsequently swell [10,26]. Gelatinization properties of rice flour were influenced by both rice variety and milling method (Table 4). These properties are mainly affected by particle size, damaged starch content, amylopectin branching, and the starch’s molecular structure [10]. Increased amylose content is linked to higher peak viscosity and rebound value. As a result, indica and japonica rice flours exhibited higher peak viscosity and rebound values compared to glutinous rice flour. The disintegration value and rebound value are commonly held to reflect the formation of the amylose gel network. However, when rice flour particles absorb water and expand upon heating, the amylose-lipid complexes within the flour exert a certain inhibitory effect on the gelatinisation process [27]. The high amylose content of japonica rice flour results in a lower peak viscosity compared to that of indica rice flour. Glutinous rice flour, which is characterized by a low amylose content, is prone to gelatinization when subjected to specific conditions. These include a lower gelatinisation temperature, disintegration value, and rebound value. The higher molecular weight of glutinous rice starch, characterized by a high number of small branches, may form a compact double-helix structure. During the process of water absorption and subsequent expansion of rice flour particles, this structure generates higher shear resistance, consequently leading to lower peak viscosity [21]. Among the milling methods, DMF resulted in the lowest peak viscosity, followed by WMF and S-DMF. S-DMF showed the highest peak viscosity, breakdown, and setback values compared to the other methods. This indicates that S-DMF starch granules swell more and are more prone to retrogradation. In contrast, DMF had the lowest peak viscosity, reflecting extensive starch damage from intense milling. WMF showed the lowest setback value, implying slower retrogradation and greater resistance to aging. Dhital et al.’s research shows that higher damaged starch and smaller starch granules mean lower peak viscosity [28].

### 3.4. Damaged Starch Content of Rice Flour

**Figure 1:**
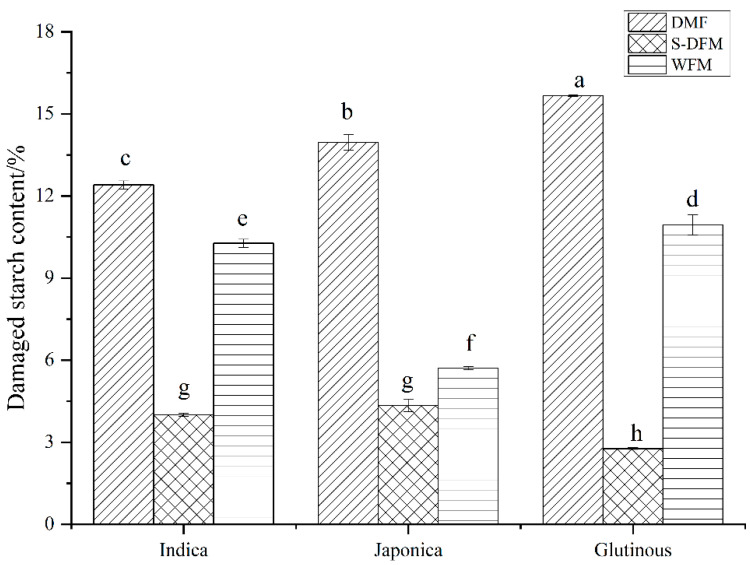
Damaged starch of different rice flours. Note: In the figure, I, J, and G denote raw materials of indica rice flour, japonica rice flour, and glutinous rice flour, respectively; DMF, S-DMF, and WMF denote dry milling, semi-dry milling, and wet milling, respectively; lowercase letters in the figure indicate significant differences between rice flours produced by different milling processes.

The main part of rice is starch. Different amounts of damaged starch can be produced by changing the milling process. This is a key way of measuring product quality as it affects the rice noodle’s texture [29]. Figure 1 compares the damaged starch content across varieties and milling methods. For any given rice variety, the DMF had the highest damaged starch content, while the S-DMF had the lowest; WMF was intermediate. This ordering reflects the intensity of mechanical stress during milling: DMF exerts the strongest friction and heat on grains, yielding the most damage, whereas pre-soaking in S-DMF and WMF softens the kernels and reduces damage. There were significant differences in damaged starch between varieties under the same milling process. This indicates that the impact of the rice variety on starch damage is modulated by the specific mechanical and thermal stresses imposed by each milling process. These findings are consistent with literature reports that DMF causes the greatest starch damage, and that WMF and S-DMF produce substantially lower damage [30].

### 3.5. Micromorphology of Rice Flour

**Figure 2:**
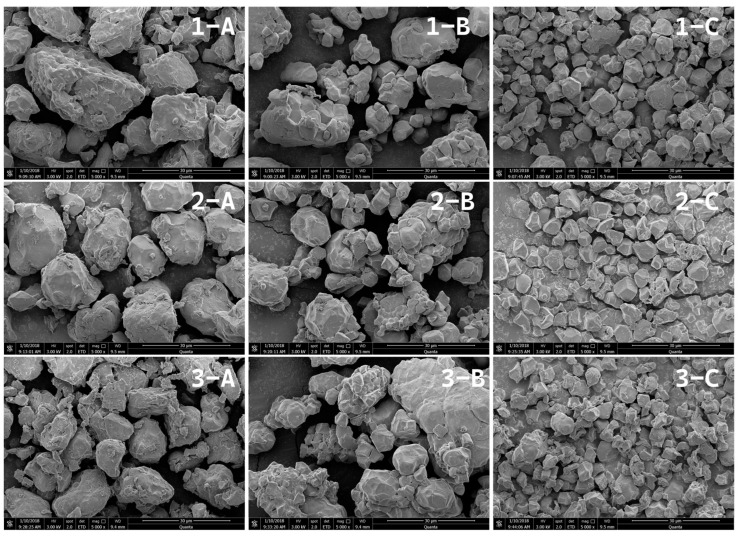
Scanning electron micrographs of flour from (**1**) indica, (**2**) japonica, and (**3**) glutinous rice processed by (**A**) dry milling, (**B**) semi-dry milling, and (**C**) wet milling.

Rice starch granules are typically 3–8 µm in size and irregularly polyhedral. SEM images (Figure 2) revealed that all rice flours consisted of angular, multifaceted particles. In the DMF japonica flour, particles were relatively uniform in size and smooth-surfaced. The DMF indica flour particles were larger and irregular, with protein fragments adhering to some granule surfaces. S-DMF showed more small particles, with some large granules partially exposed and fused with fines. WMF contained only small, sharp-edged granules distributed uniformly; notably, glutinous WMF exhibited some granule aggregation. These observations agree with the particle size data. They also align with Lorlowhakarn and Naivikul [31], who noted that wet milling preserves starch crystalline structure, as seen here by the well-defined granule edges.

### 3.6. Solubility and Swelling

Solubility and swelling power reflect the strength of starch-water interactions during gelatinization and the ability of flour to retain water under centrifugal force [32]. As shown in Table 5, the solubility of all rice flours increased with rising temperature until a maximum was reached, after which it decreased. Glutinous rice flour exhibited the highest solubility, with both DMF and WMF glutinous flours continuing to increase in solubility as the temperature rose. Among the different milling processes, S-DMF exhibited significantly lower solubility than WMF and DMF. Solubility is strongly influenced by damaged starch content. When rice flour is not fully heated, dissolution is driven by amylopectin; as milling intensity increases, amylopectin degradation is enhanced, making it more soluble in water [33]. Therefore, the high solubility of glutinous rice flour in water is related to its elevated damaged-starch level and higher amylopectin content. Similarly, the low damaged-starch content of S-DMF corresponds to its lower solubility. The initial increase in solubility with temperature is primarily due to hydrogen-bond rupture and the transition of starch crystalline regions into amorphous domains; during this process, linear amylose present in crystalline regions and within starch–lipid complexes begins to leach out [34,35], However, excessive heating in aqueous media can irreversibly destroy the ordered starch molecular architecture and induce protein denaturation and cross-linking, which reduce solubility. Consequently, solubility declines at excessively high temperatures [36].

As shown in Table 6, swelling power and solubility exhibit similar trends: except for G-WMF, swelling power increased with temperature to a peak and then decreased for all flours, with DMF showing the highest swelling power. This behavior is mainly attributable to progressive water uptake and swelling of flour and starch granules with increasing temperature, accompanied by starch solubilization and increased solubility; at very high temperatures, starch granules swell irreversibly and collapse, disrupting their molecular structure and reducing swelling power [37]. Analogous to solubility, swelling power correlates positively with damaged-starch content because damaged starch enhances water uptake and hydration characteristics. Because DMF imparts greater mechanical damage, DMF is more prone to water absorption and swelling [15,38].

Solubility and swelling power characterize starch and water interactions during heating. The solubility of S-DMF was found to be significantly lower than that of DMF and WMF at both 40 °C and 80 °C (*p* < 0.05), as shown in Table 4 and Table 5. For DMF and S-DMF, solubility increased with temperature up to about 60–70 °C, then declined at higher temperatures. Glutinous flours exhibited the highest solubility overall, consistent with their higher amylopectin content. The swelling power of DMF was significantly higher than that of S-DMF and WMF at 40 °C and 60 °C. This trend is associated with starch damage; greater damage leads to more amylopectin leaching, resulting in higher solubility and swelling at moderate temperatures. In our study, the low damaged starch content in S-DMF resulted in its lower solubility and swelling. At high temperatures, solubility and swelling decreased for all samples, likely due to the disruption of starch crystallinity, irreversible granule collapse, and protein network formation [38,39].

### 3.7. Relative Crystallinity of Rice Flour

**Figure 3:**
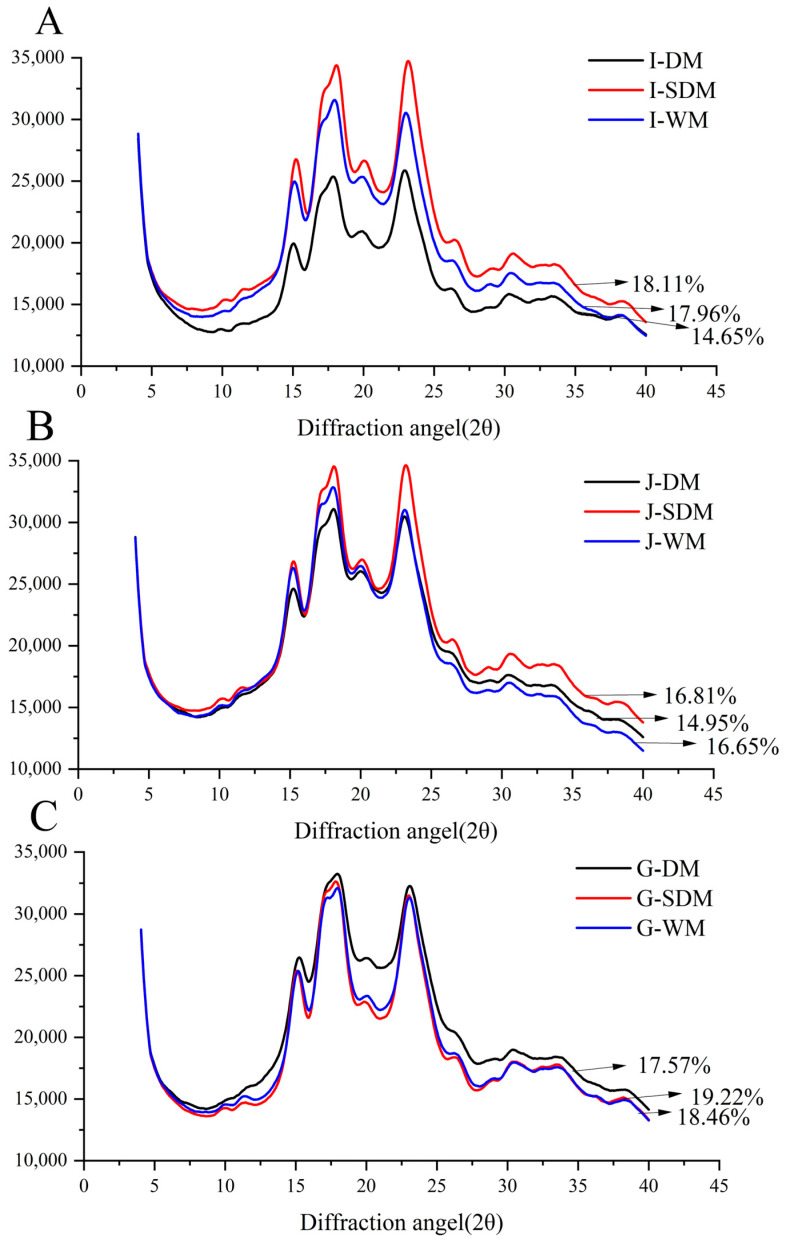
Crystallinity characteristics of different rice flours. Note: In the diagram, (**A**–**C**) represent indica rice flour, japonica rice flour, and glutinous rice flour, respectively.

The XRD patterns for all rice flour samples, as shown in Figure 3, exhibited the characteristic peaks of an A-type starch crystalline structure, with distinct reflections at 2θ angles of approximately 15°, 18°, 20°, and 23°. This pattern is typical for cereal starches, including rice. The presence of these sharp peaks confirms that the inherent crystalline order of the starch granules was maintained across all samples, regardless of the milling method or rice variety. Typically, materials with higher relative crystallinity produce sharper, narrower diffraction peaks [38,40]. In this study, DMF showed lower diffraction peak intensities, indicating a reduction in crystalline order. This loss of crystallinity is attributed to the intense mechanical forces during dry milling, which cause granule fragmentation and degradation of the ordered crystalline structure. As a result, damaged starch increases and crystalline regions are converted to amorphous phases [41,42]. In contrast, S-DMF exhibited the highest relative crystallinity among the treatments, likely due to the tempering step in the semi-dry process, which reduces mechanical damage, preserves granule integrity, and limits the formation of damaged starch. These XRD observations align with the microstructural features observed in Figure 2.

### 3.8. Thermal Properties of Rice Flour

DSC measures the enthalpic changes during starch gelatinization, reflecting the transition from semi-crystalline to amorphous starch. The DSC gelatinization parameters are summarized in Table 7. Glutinous rice flours showed significantly lower onset, peak, and conclusion temperatures (T0, Tp, Tc) compared to indica and japonica flours. This finding is consistent with Jane et al.’s observations, which noted that glutinous starches have the shortest average branch chain length, resulting in the lowest pasting temperature [27]. Within each variety, milling methods had minimal effects on T0, Tp, or Tc. Generally, flours from dry and semi-dry milling had slightly higher Tp and Tc, likely due to their larger average particle size, which provides greater thermal resistance [43]. In contrast, the gelatinization enthalpy (ΔH) was higher for S-DMF and WMF. ΔH is positively correlated with crystallinity and negatively correlated with damaged starch content [10]. The low damaged starch content and high crystallinity in S-DMF and WMF samples require more energy to melt, resulting in higher ΔH values.

### 3.9. Rheological Properties of Rice Flour

**Figure 4:**
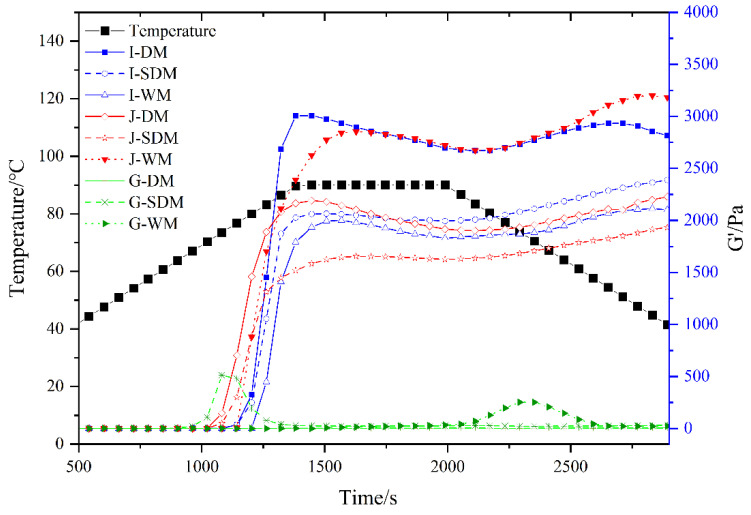
Temperature sweep of different rice flours.

Conducting a rheological analysis of rice flour paste and investigating its dynamic rheological characteristics as a function of temperature is imperative. Rheological analysis (Figure 4, Table 8) revealed the temperature-dependent viscoelastic behavior of rice starch flour. Initially, as samples were heated, the storage modulus (G′) remained relatively constant. However, between 80 and 90 °C, G′ increased sharply as starch gelatinization occurred. Glutinous rice flour exhibited much lower G′ and a higher loss factor (tanδ) than indica and japonica flour, indicating a more viscous nature. Notably, dry-milled glutinous flour did not show a clear G′ₘₐₓ peak. This behavior likely stems from glutinous rice’s high amylopectin content. Both indica and japonica rice flours reached a maximum storage modulus (G′ₘₐₓ) at around 90 °C, with G′ showing a slight increase during subsequent cooling. This behavior can be explained by the progressive water uptake and swelling of starch granules upon heating, followed by the sequential leaching of amylopectin starch molecules; released chains entangle and form a cohesive network, thereby increasing both G′ and G″ [44]. However, when heated to 80–90 °C, the solubility and swelling power of the flours attain their maxima, after which granule rupture commences, and G′ begins to decline. During cooling, starch retrogradation proceeds and hydrogen bonds reform, leading to a modest recovery in G′. S-DMF exhibited the lowest onset temperature for the increase in G′, likely due to its larger particle size, which facilitates earlier water uptake and swelling.

## 4. Conclusions

This study comprehensively evaluated the effects of three rice milling methods, with dry, semi-dry, and wet processes, on three types of broken rice (indica, japonica, and glutinous rice). The results indicate that milling methods significantly affect the physicochemical properties of rice flour, with distinct variations observed between the rice varieties. Dry milling led to the highest degree of starch damage, which resulted in increased solubility and swelling power, but it also caused a reduction in gelatinization temperature and paste viscosity, negatively affecting the overall quality of the rice flour. Semi-dry milling, on the other hand, offered a balanced approach, showing moderate starch damage and enhanced thermal stability compared to dry milling. This method preserved the desirable functional properties of rice flour, making it more suitable for products requiring higher stability and texture. Wet milling, while preserving starch integrity and minimizing damage, produced finer particles with lower swelling power and solubility, limiting its application in certain food products. Additionally, Japonica rice showed less susceptibility to milling-induced damage, maintaining better functional properties compared to Indica and Glutinous rice. The study emphasizes the importance of selecting the appropriate milling method based on the desired characteristics of rice flour, especially in the production of gluten-free rice-based products. Future research should focus on optimizing semi-dry milling processes to enhance their scalability and sustainability in industrial applications, particularly for gluten-free food manufacturing.

## Figures and Tables

**Table 1:** Physicochemical indices of different rice.

Sample Name	Moisture Content (%, Dry Basis)	Ash Content (%, on a Dry Basis)	Protein (%, Dry Basis)	Amylose Content (%, Dry Basis)
Indica	10.60 ± 0.06 ^b^	0.38 ± 0.03 ^ab^	8.62 ± 0.03 ^a^	16.2 ± 0.4 ^a^
Japonica	10.96 ± 0.04 ^a^	0.42 ± 0.01 ^a^	7.85 ± 0.01 ^b^	16.4 ± 0.2 ^a^
Glutinous	9.43 ± 0.07 ^c^	0.35 ± 0.02 ^b^	6.88 ± 0.07 ^c^	4.0 ± 0.0 ^b^

Note: Lowercase letters denote significant differences between rice noodles produced by distinct milling processes; the same applies below.

**Table 2:** Physicochemical indices of rice flours produced by different milling processes.

Sample	Water/%	Ash/%	Whiteness	Protein/%	Starch/%
I-DMF	10.60 ± 0.06 ^c^	0.38 ± 0.03 ^bc^	86.10 ± 0.00 ^f^	8.62 ± 0.03 ^b^	90.73 ± 0.17 ^b^
I-S-DMF	10.60 ± 0.13 ^c^	0.39 ± 0.01 ^ab^	81.50 ± 0.00 ^h^	8.61 ± 0.02 ^b^	89.35 ± 0.09 ^c^
I-WMF	7.00 ± 0.00 ^g^	0.29 ± 0.00 ^ef^	87.25 ± 0.07 ^d^	8.76 ± 0.07 ^a^	86.82 ± 0.08 ^f^
J-DMF	10.96 ± 0.04 ^b^	0.42 ± 0.01 ^a^	89.30 ± 0.00 ^c^	7.85 ± 0.01 ^d^	90.37 ± 0.17 ^b^
J-S-DMF	11.90 ± 0.08 ^a^	0.40 ± 0.01 ^ab^	86.00 ± 0.00 ^g^	8.46 ± 0.04 ^c^	88.77 ± 0.69 ^c^
J-WMF	7.11 ± 0.10 ^g^	0.27 ± 0.01 ^f^	90.60 ± 0.00 ^b^	7.93 ± 0.02 ^d^	87.09 ± 0.33 ^f^
G-DMF	9.43 ± 0.07 ^f^	0.35 ± 0.02 ^cd^	90.60 ± 0.00 ^b^	6.88 ± 0.07 ^f^	93.01 ± 0.17 ^a^
G-S-DMF	10.32 ± 0.15 ^d^	0.33 ± 0.01 ^de^	86.30 ± 0.00 ^e^	7.64 ± 0.08 ^e^	92.78 ± 0.08 ^a^
G-WMF	9.84 ± 0.05 ^e^	0.17 ± 0.03 ^g^	91.30 ± 0.00 ^a^	6.98 ± 0.03 ^f^	87.88 ± 0.42 ^d^

Note: In the table, I, J, and G denote raw materials of indica rice flour, japonica rice flour, and glutinous rice flour, respectively; DMF, S-DMF, and WMF denote dry milling, semi-dry milling, and wet milling, respectively. Values are mean ± SD (*n* = X). Means with different letters within the same column differ significantly (*p* < 0.05).

**Table 3:** Particle diameter distribution of different rice flours with different milling processes.

Sample	D10/µm	D50/µm	D90/µm
I-DMF	4.09 ± 0.02 ^a^	16.51 ± 0.25 ^b^	43.32 ± 0.76 ^b^
I-S-DMF	3.93 ± 0.39 ^a^	19.50 ± 1.56 ^a^	50.06 ± 5.09 ^a^
I-WMF	2.00 ± 0.10 ^cd^	8.04 ± 0.19 ^e^	25.84 ± 2.50 ^d^
J-DMF	3.12 ± 0.05 ^b^	9.11 ± 0.38 ^de^	22.87 ± 1.04 ^d^
J-S-DMF	2.24 ± 0.01 ^c^	10.31 ± 0.00 ^d^	37.26 ± 0.50 ^c^
J-WMF	1.93 ± 0.05 ^cd^	8.13 ± 0.04 ^e^	23.86 ± 0.02 ^d^
G-DMF	2.02 ± 0.01 ^cd^	7.86 ± 0.04 ^e^	22.81 ± 0.41 ^d^
G-S-DMF	2.02 ± 0.16 ^cd^	12.05 ± 1.48 ^c^	37.53 ± 4.69 ^c^
G-WMF	1.70 ± 0.01 ^d^	7.56 ± 0.58 ^e^	24.82 ± 0.34 ^d^

Note: D10, D50, and D90 indicate the cumulative particle size distributions of the samples, respectively, at 10%, 50%, and 90%. Values are mean ± SD (n = X). Means with different letters within the same column differ significantly (*p* < 0.05).

**Table 4:** Brabender viscosity of different rice flours with different milling processes.

Sample	Pasting Temperature (°C)	Peak Viscosity (BU)	Breakdown (BU)	Setback (BU)
I-DMF	67.6	839	562	197
I-S-DMF	69.1	912	600	235
I-WMF	69.3	857	559	223
J-DMF	62.9	560	386	142
J-S-DMF	65.6	736	477	197
J-WMF	66.3	640	455	128
G-DMF	56.1	117	79	16
G-S-DMF	59.1	258	200	25
G-WMF	60.1	399	314	35

**Table 5:** Solubility of different rice flours.

Sample	50 °C	60 °C	70 °C	80 °C	90 °C
I-DMF	2.81 ± 0.09 ^e^	2.54 ± 0.11 ^c^	2.77 ± 0.10 ^d^	2.78 ± 0.08 ^de^	3.21 ± 0.22 ^c^
I-S-DMF	1.41 ± 0.02 ^g^	1.43 ± 0.16 ^c^	1.55 ± 0.02 ^d^	2.33 ± 0.13 ^e^	1.67 ± 0.16 ^c^
I-WMF	1.67 ± 0.16 ^c^	3.43 ± 0.01 ^c^	3.49 ± 0.01 ^cd^	3.22 ± 0.04 ^de^	3.20 ± 0.18 ^c^
J-DMF	3.40 ± 0.03 ^d^	3.35 ± 0.01 ^c^	3.66 ± 0.02 ^cd^	6.01 ± 0.77 ^cd^	3.11 ± 0.14 ^c^
J-S-DMF	1.35 ± 0.01 ^g^	1.43 ± 0.00 ^c^	1.50 ± 0.09 ^d^	7.71 ± 0.60 ^c^	1.73 ± 0.02 ^c^
J-WMF	2.85 ± 0.04 ^e^	2.98 ± 0.04 ^c^	2.85 ± 0.14 ^d^	2.75 ± 0.16 ^de^	2.75 ± 0.02 ^c^
G-DMF	14.99 ± 0.12 ^a^	16.79 ± 0.08 ^a^	25.50 ± 1.96 ^a^	45.08 ± 1.12 ^a^	46.22 ± 4.74 ^a^
G-S-DMF	1.84 ± 0.04 ^f^	2.88 ± 0.13 ^c^	5.42 ± 0.96 ^c^	20.44 ± 4.06 ^b^	9.80 ± 2.11 ^b^
G-WMF	7.62 ± 0.04 ^b^	9.73 ± 0.28 ^b^	15.49 ± 1.61 ^b^	19.23 ± 0.34 ^b^	45.59 ± 0.72 ^a^

Values are mean ± SD (*n* = X). Means with different letters within the same column differ significantly (*p* < 0.05).

**Table 6:** Swelling power of different rice flours.

Sample	50 °C	60 °C	70 °C	80 °C	90 °C
I-DMF	2.99 ± 0.02 ^b^	3.17 ± 0.06 ^c^	3.39 ± 0.04 ^e^	10.38 ± 0.42 ^bc^	8.69 ± 0.35 ^bc^
I-S-DMF	2.50 ± 0.04 ^e^	2.68 ± 0.02 ^e^	2.99 ± 0.08 ^f^	10.97 ± 0.54 ^bc^	8.85 ± 0.11 ^b^
I-WMF	2.73 ± 0.00 ^c^	2.77 ± 0.05 ^e^	3.05 ± 0.00 ^f^	8.04 ± 0.21 ^de^	8.08 ± 0.04 ^d^
J-DMF	3.37 ± 0.03 ^a^	3.95 ± 0.05 ^a^	4.08 ± 0.03 ^c^	12.43 ± 1.88 ^b^	8.25 ± 0.13 ^cd^
J-S-DMF	2.56 ± 0.01 ^e^	2.71 ± 0.05 ^e^	4.00 ± 0.10 ^cd^	12.47 ± 1.73 ^b^	8.64 ± 0.31 ^bc^
J-WMF	2.71 ± 0.01 ^c^	2.97 ± 0.03 ^d^	3.75 ± 0.02 ^d^	7.68 ± 0.35 ^de^	7.48 ± 0.23 ^e^
G-DMF	3.35 ± 0.04 ^a^	3.73 ± 0.19 ^b^	5.34 ± 0.14 ^b^	15.33 ± 0.03 ^a^	8.50 ± 0.04 ^bcd^
G-S-DMF	2.71 ± 0.04 ^c^	2.83 ± 0.01 ^de^	5.50 ± 0.27 ^b^	8.89 ± 1.17 ^cd^	5.38 ± 0.32 ^f^
G-WMF	2.64 ± 0.01 ^d^	2.79 ± 0.02 ^e^	7.67 ± 0.03 ^a^	6.40 ± 0.19 ^e^	10.14 ± 0.15 ^a^

Values are mean ± SD (*n* = X). Means with different letters within the same column differ significantly (*p* < 0.05).

**Table 7:** DSC parameters of different rice flours.

Sample	T_0_/°C	T_P_/°C	T_C_/°C	ΔH/(J/g)
I-DMF	66.56 ± 1.17 ^a^	73.41 ± 0.21 ^a^	83.94 ± 0.69 ^a^	6.56 ± 1.51 ^bc^
I-S-DMF	64.88 ± 0.07 ^a^	72.13 ± 0.46 ^a^	83.70 ± 0.11 ^a^	10.00 ± 0.13 ^a^
I-WMF	65.24 ± 0.23 ^a^	72.74 ± 0.15 ^a^	83.15 ± 0.61 ^a^	7.97 ± 0.42 ^ab^
J-DMF	61.58 ± 0.36 ^b^	69.33 ± 0.05 ^b^	79.64 ± 0.14 ^b^	6.33 ± 0.39 ^bc^
J-S-DMF	61.32 ± 0.06 ^b^	68.75 ± 0.18 ^b^	78.95 ± 1.13 ^b^	9.10 ± 1.15 ^a^
J-WMF	61.97 ± 0.33 ^b^	68.58 ± 0.26 ^b^	77.39 ± 0.84 ^bc^	6.56 ± 1.64 ^bc^
G-DMF	60.68 ± 1.18 ^bc^	67.68 ± 0.42 ^bc^	75.69 ± 0.91 ^c^	4.71 ± 0.44 ^c^
G-S-DMF	58.43 ± 0.59 ^c^	66.32 ± 0.01 ^c^	75.42 ± 0.21 ^c^	8.38 ± 0.05 ^ab^
G-WMF	60.87 ± 2.38 ^b^	66.89 ± 1.90 ^c^	77.52 ± 2.32 ^bc^	8.78 ± 0.41 ^a^

Values are mean ± SD (*n* = X). Means with different letters within the same column differ significantly (*p* < 0.05).

**Table 8:** Temperature sweep parameters of different rice flours.

Sample	T_G′_/°C	G′_max_/Pa	T_G′max_/°C	G′25 °C/Pa	tan25 °C
I-DMF	76.75	3007	89.66	2713	0.0735
I-S-DMF	73.53	2024	90.01	2476	0.0736
I-WMF	79.98	2001	90.01	1983	0.0647
J-DMF	73.53	2164	86.40	2350	0.0594
J-S-DMF	70.29	1654	90.01	2045	0.0631
J-WMF	76.75	2854	90.01	3039	0.0400
G-DMF	86.40	9	25.00	9	0.8600
G-S-DMF	63.79	478	76.76	45	0.5104
G-WMF	90.10	252	70.75	27	0.5205

Note: T_G′_ denotes the temperature at which G′ begins to rise; G′_max_ represents the maximum value attained during the first G′ rise; T_G′max_ denotes the temperature corresponding to G′_max_; G′25 °C indicates the G′ value corresponding to cooling to 25 °C; tan25 °C denotes the loss factor value at 25 °C.

## Data Availability

The original contributions presented in the study are included in the article; further inquiries can be directed to the corresponding authors.

## Abbreviations

The following abbreviations are used in this manuscript:

I	Indica
J	Japonica
G	Glutinous rice
I-DMF	Indica dry-milled flour
J-DMF	Japonica dry-milled flour
G-DMF	Glutinous dry-milled flour
I-S-DMF	Indica semi-dry-milled flour
J-S-DMF	Japonica semi-dry-milled flour
G-S-DMF	Glutinous semi-dry-milled flour
I-WMF	Indica wet-milled flour
J-WMF	Japonica wet-milled flour
G-WMF	Glutinous wet-milled flour
XRD	X-ray diffraction
